# Mean Platelet Volume and Arterial Stiffness – Clinical Relationship and Common Genetic Variability

**DOI:** 10.1038/srep40229

**Published:** 2017-01-06

**Authors:** Marina Panova-Noeva, Natalie Arnold, M. Iris Hermanns, Jürgen H. Prochaska, Andreas Schulz, Henri M. Spronk, Harald Binder, Norbert Pfeiffer, Manfred Beutel, Stefan Blankenberg, Tanja Zeller, Johannes Lotz, Thomas Münzel, Karl J. Lackner, Hugo ten Cate, Philipp S. Wild

**Affiliations:** 1 Center for Thrombosis and Hemostasis (CTH), University Medical Center of the Johannes Gutenberg-University Mainz, Germany; 2 Center for Cardiology I, University Medical Center of the Johannes Gutenberg-University Mainz, Germany; 3 Preventive Cardiology and Preventive Medicine, Center for Cardiology, University Medical Center of the Johannes Gutenberg-University Mainz, Germany; 4 Laboratory for Clinical Thrombosis and Hemostasis, Department of Internal Medicine, Cardiovascular Research Institute Maastricht (CARIM), Maastricht University Medical Center, The Netherlands; 5 Institute of Medical Biostatistics, Epidemiology and Informatics, University Medical Center of the Johannes Gutenberg-University Mainz, Germany; 6 Department of Ophthalmology, University Medical Center of the Johannes Gutenberg-University Mainz, Germany; 7 Department of Psychosomatic Medicine and Psychotherapy, University Medical Center of the Johannes Gutenberg-University Mainz, Germany; 8 Department of General and Interventional Cardiology, University Heart Centre, Hamburg-Eppendorf, Germany; 9 DZHK (German Center for Cardiovascular Research), Partner Site Hamburg/Kiel/Lübeck, Hamburg, Germany; 10 Institute for Clinical Chemistry and Laboratory Medicine, University Medical Center of the Johannes Gutenberg-University Mainz, Mainz, Germany; 11 DZHK (German Center for Cardiovascular Research), Partner Site RhineMain, Mainz, Germany

## Abstract

Vessel wall stiffening is an important clinical parameter, but it is unknown whether platelets, key elements in the pathogenesis of arterial thrombosis, are associated with arterial stiffness. The present studies sought to determine whether mean platelet volume (MPV), a potential marker of platelet activation, is linked to vascular elasticity as assessed by the augmentation index (AIx), in 15,010 individuals from the population-based Gutenberg Health Study. Multivariable analysis showed that MPV in both males (β 0.776; 95^th^CI [0.250;1.16]; p = 0.0024) and females (β 0.881[0.328;1.43]; p = 0.0018) is strongly associated with AIx. Individuals with MPV and AIx above the sex-specific medians had worse survival. Association analysis between MPV-related genetic variants and arterial stiffness identified four genetic variants in males and one in females related with AIx. Cox regression analysis for mortality identified one of these joint genetic variants close to *ring finger protein 145* gene (*RNF145*, rs10076782) linked with increased mortality (hazard ratio 2.02; 95^th^CI [1.35;3.02]; p = 0.00061). Thus, these population-based data demonstrate a close relation between platelet volume as a potential marker of platelet activation and arterial stiffness in both sexes. Further research is warranted to further elucidate the mechanisms underlying larger platelets‘ role in arterial stiffening including the role of shared common genetics.

Considering the high morbidity and mortality burden triggered by atherosclerosis, efforts are clearly directed towards early recognition and diagnosis of subclinical atherosclerosis. Arterial stiffness as a measure of distensibility and compliance of vessels is an established marker of cardiovascular morbidity and mortality and therefore a potential therapeutic target^1^,^2^. Arterial hypertension and aging are established factors contributing to arterial stiffness and the role of inflammation in decreasing vascular compliance is becoming more evident as well^3^,^4^.

The role of platelets in causing increased vascular stiffness is much less well defined^5^,^6^,^7^,^8^,^9^. Activated platelets release a numerous number of substances that control vascular permeability, regulate vasoconstriction or vasodilatation, stimulate macrophage recruitment and infiltration, release reactive oxygen species (ROS) and stimulate ROS generation within the vascular wall leading to decreased vascular nitric oxide (NO) bioavailability^10^. Therefore, it is of outmost importance to better understand the interaction between arterial stiffness and parameters of platelet activation such as mean platelet volume (MPV) with regard to underlying cellular, molecular and genetic mechanisms and their clinical impact.

Several studies suggest that vessel wall stiffening is an important clinical target, but it is unknown to what extent platelets, key elements in the pathophysiology of arterial thrombosis, are associated with arterial stiffness. The Gutenberg Health Study (GHS), as a large, prospective population-based cohort study in Western Germany, offers the potential to explore this relation in depth. To the best of our knowledge, this is the first study to investigate the relation between mean platelet volume (MPV), a potential marker of platelet activation including its known genetic variants, and vascular stiffness, in a large adult general population study sample.

## Methods

### Population sample

Arterial stiffness and MPV have been determined in 15,010 participants enrolled in the GHS at a baseline examination in the period between April 2007 and April 2012. GHS is a population-based, prospective, observational, single-center cohort study in Western Mid-Germany designed predominantly for examination of cardiovascular disease. Residents of the City of Mainz and the County Mainz-Bingen aged 35 to 74 were selected randomly from the local governmental registry offices with a stratification for sex, residence (urban vs. rural), and decade of age. All participants underwent a 5-hour examination at the study center following standard operating procedures (SOPs) by certified medical technical assistants. Details of the study design have been published earlier^11^. The study protocol was approved by the Ethics Committee of the State Chamber of Physicians of Rhineland-Palatinate, Germany (Ref.No. 837.020.07(5555)) and the study project was approved by the steering committee of the Gutenberg Health Study. The study has been conducted in accordance with the Declaration of Helsinki. Written informed consent was obtained from all participants before entering the study.

### Assessment of arterial stiffness

Arterial stiffness was assessed by digital volume plethysmography using an EndoPat 2000 device (Itamar Medical, Caesarea, Israel), which is based on the fingertip measurement of pulsatile volume changes. The resultant augmentation index (AIx) is automatically calculated by identifying the early (P1) and late systolic peak (P2) with the following formula: (P2-P1)/P1*100. Endo-PAT 2000 has been already validated in many studies for both arterial stiffness and endothelial function measurements^12^,^13^,^14^,^15^,^16^.

### Blood sampling and laboratory measurements

Venous blood sampling was performed using tubes containing K3-ethylenediaminetetraacetic acid (EDTA). Platelet parameters (count and MPV) have been automatically determined on an Advia 120 Hematology System (Siemens, Germany). Coefficients of variation for MPV range between 0.8 and 2.2% over time with a mean of 1.4%. Details of internal quality control for Advia 120 hematology systems and validation for inter-instrument variation for MPV measurements have been published earlier^17^. Biochemical analysis of blood glucose and C-reactive protein measured in plasma and total cholesterol, high density lipoprotein (HDL), and triglycerides measured in serum were determined within 1 hour after sampling by routine methods. Low density lipoprotein (LDL) cholesterol was calculated by Friedewald´s formula. All laboratory measurements have been performed in the central laboratory of University Medical Center Mainz.

### Genotyping and imputation of Single Nucleotide Polymorphisms (SNPs)

From the genetic data available for study participants, we systematically searched and selected 51 SNPs (see Supplemental Table 1) which had been described by large genome wide association studies (GWAS) for MPV to test them for association with arterial stiffness^18^,^19^,^20^,^21^. For the available genetic information, genotyping was conducted on the Affymetrix Genome-Wide Human SNP 6.0 array (Affymetrix, Santa Clara, CA) according to the manufacturer´s recommendations. After quality control, genetic data were available for analysis in 4,175 individuals. The methodology for genotyping has been described before^17^.

### Statistical analysis

Variable of arterial stiffness derived from digital plethysmography was Augmentation Index (AIx) expressed as percentage augmentation (%). Distribution of MPV is presented as median with interquartile range and tested with the Mann–Whitney U test. Normally distributed continuous variables are described using mean ± standard deviation (SD) and tested with T-test, whereas categorical variables are displayed by absolute and relative frequency and tested by Chi-squared test if needed. The reference group is a subsample of the population sample and included 2,660 individuals without manifest disease (i.e. myocardial infarction, coronary artery disease, stroke, peripheral arterial disease, venous thromboembolism, atrial fibrillation, chronic heart failure, cancer, kidney disease, liver disease, chronic obstructive pulmonary disease and anemia) and no known traditional cardiovascular risk factors (i.e. hypertension, obesity, dyslipidemia, diabetes mellitus, smoking and positive family history (FH) for myocardial infarction (MI) or stroke). Relations between continuous variables were explored by Pearson´s correlation coefficients. Multivariable linear regression analysis with AI as dependent variable and adjustment for age, cardiovascular risk factors (CVRFs), comorbidities and medication was used to identify determinants of arterial stiffness. The analysis of prospective mortality data with censoring is presented by using plots of Kaplan Meier curves with log-rank test and by Cox proportional hazards models.

Because of the explorative character of the analysis, a significance threshold was not defined for p-values. P-values should be interpreted as a continuous measure of statistical evidence.

## Results

### Arterial stiffness and MPV

**Figure 1:**
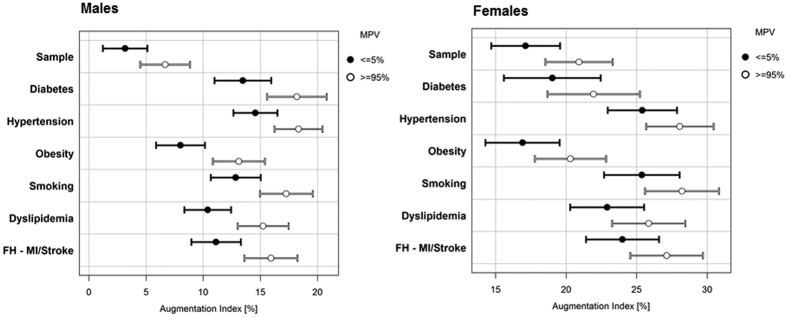
Arterial stiffness in the population sample without and subsamples with cardiovascular risk factors. Forest plot presenting the mean (95% CI) of augmentation index in both, males and females estimated for the sample ≤5^th^ and ≥95^th^ percentile of MPV. FH-MI/Stroke, Family history of myocardial infarction or stroke.

Arterial stiffness in the population sample without traditional CVRFs and in subsamples with CVRFs is presented sex-specifically in Fig. 1. Presented AIx values are estimated for the subgroups below the 5^th^% and above 95^th^% of MPV. In general, individuals with CVRFs had higher AIx compared to the sample without CVRFs. Males with a CVRF had higher AIx. Differently, in females, only those with hypertension, smoking, dyslipidemia and positive FH for MI or stroke had higher AIx with respect to the sample without CVRFs.

**Figure 2:**
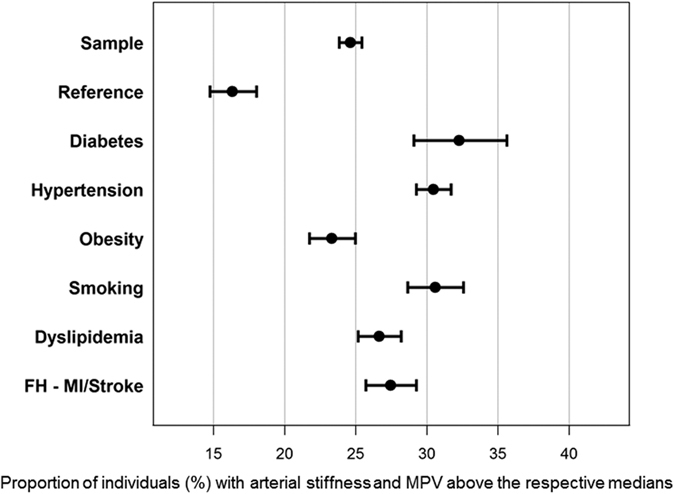
Proportion of individuals with both MPV and arterial stiffness above the median in subgroups with and without cardiovascular risk factors (CVRFs). Forest plot presenting the relative frequency with 95% CI of individuals in each subgroup having augmentation index and mean platelet volume above the sex-specific medians. AIx, augmentation index; MPV, mean platelet volume; FH-MI/Stroke, family history of myocardial infarction or stroke.

As shown in Table 1, AIx increased with increasing MPV quartiles in both males and females. When stratifying the population sample for sex-specific medians of MPV (males: 8.4 fL; females 8.5 fL) and AIx (males: 8.90%; females: 20.6%) as cut-off values, approx. 25% of the population sample had AIx and MPV with both parameters above the respective median value (Fig. 2). Within individuals with CVRFs, most subgroups had 30% and more of individuals with these characteristics. The highest proportion was observed for individuals with diabetes mellitus with 35% (95% CI: 32%; 38%) having AIx and MPV above the medians.

The unadjusted and the multivariable linear regression model for arterial stiffness, with adjustment for age, CVRFs, comorbidities, medication, fibrinogen and hematocrit confirmed the association between higher MPV and AIx for both males and females (Table 2). Adjusting the model of AIx in females additionally for the hormonal status (i.e. intake of oral contraceptives, hormone replacement therapy and presence of menstrual bleeding) did not change the observed association between MPV and AIx (β estimate: 0.968 [0.39; 1.54], p = 0.00096). Differently, as shown in the Table 2, platelet count was weakly associated to AIx for both sexes in a crude analysis and the association was lost in the multivariable adjusted models.

### Association between MPV related SNPs and arterial stiffness

Linear regression models for the associations between MPV related SNPs and AIx in males are presented in Table 3. Of the 51 SNPs associated with MPV, 4 SNPs showed an association with AIx in males. Three SNPs showed a positive association (rs7342306, rs10076782 and rs17397129) and 1 SNP (rs7961894) a negative association with AIx. After adjusting for age and CVRFs the association remained for rs17397129 (β estimate: 1.60 [0.114; 3.08], p = 0.035). At the next step, further adjusting for comorbidities and medication no association remained significant. In females, rs11734132 was inversely associated to AIx both in the crude and adjusted analysis for age and CVRFs (β estimate: −1.92 [−3.61; −0.217], p = 0.027). When further adjusting for comorbidities, the association was no longer present. The full association analysis is presented in Supplemental Table 2. Overall, all associations found were of moderate nature.

### Interaction of MPV, arterial stiffness and MPV-related SNPs to survival

**Figure 3:**
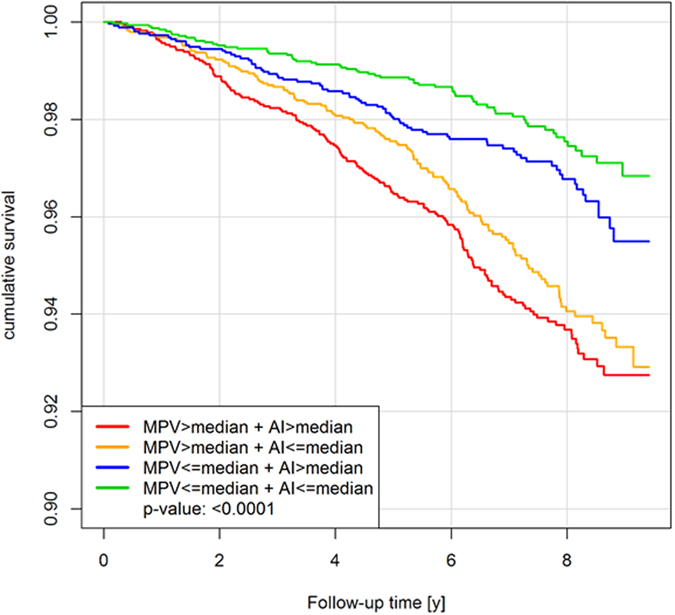
The effect of mean platelet volume and arterial stiffness on survival. Kaplan Meier curves for cumulative survival depending on mean platelet volume and arterial stiffness in the overall sample with N = 14,580. Individuals were stratified for the sex-specific medians of mean platelet volume and augmentation index as cut-off values.

During a follow-up period until September 2016 with a median follow-up time of 6.8 [5.5–8.2] years, a total of 580 deaths were registered in the total sample. When stratifying individuals for the sex-specific medians of both MPV and AIx, the best survival was present for the combination of both MPV and AIx below the respective medians (Fig. 3). Cox regression analysis modeling for death including MPV, the interaction term MPV*AIx, and with adjustment for age and sex, heart rate and height, confirmed the association for both MPV and AIx with increased total mortality (Supplemental Table 3). All identified MPV-related SNPs that were related with AIx were tested for an association with total mortality (Table 4). The results of an age and sex adjusted cox-regression model revealed one genetic variant close to *ring finger protein 145* gene (*RNF145,* rs10076782) significantly associated with increased mortality (hazard ratio, 2.02; 95% CI, 1.35 to 3.02; p = 0.00061).

## Discussion

To our knowledge, this is a largest population-based study investigating the association between MPV, a potential marker of platelet activity and arterial stiffness. With this study, in a multivariable analysis even after adjustment, we found that there is a positive, strong association between MPV and vascular stiffness in both men and women. An association analysis identified one genetic variant close to *ring finger protein 145* gene (*RNF145*, rs10076782) linked with increased mortality.

### MPV and vascular stiffness, biomarker for cardiovascular outcomes

MPV has been previously examined as an index of platelet function and associated to other conventional markers of platelet activation^22^,^23^,^24^. It is known that larger platelets contain more granules and are in general enzymatically and metabolically more active compared to smaller ones exhibiting as a consequence more pro-thrombotic characteristics. Larger platelets secrete more serotonin and beta thromboglobulin and produce more thromboxane A2 than smaller platelets, leading to more vascular complications^25^.

Vascular stiffness as assessed by the determination of the augmentation index is well validated in large population studies to be a strong predictor of adverse cardiovascular outcomes^26^,^27^, and has been incorporated into the current guideline for the management of arterial hypertension as a searching tool for subclinical organ damage^28^. Since, MPV has been also shown to be associated with increased risk of arterial and venous thrombosis and recent studies indicate that MPV is significantly determined by traditional CVRFs and a higher MPV goes along with a higher risk of death^17^, it is tempting to conclude that increased MPV may adversely affect vascular function (stiffness) mainly because of its functional “side effects”.

Indeed, the main finding of the present analysis is a positive and strong association between MPV and arterial stiffness, implicating a close link between higher MPV, as potential marker of platelet activation, and increased stiffness of the arterial vessel wall. Differently, platelet count was not associated to arterial stiffness, as previously reported^29^. With the present study we could also establish that both, a higher MPV as well as increased vascular arterial stiffness are associated with worse survival. Higher MPV and increased arterial stiffness were present in individuals with present CVRFs, particularly diabetes, hypertension and smoking. The finding did not differ between males and females, suggesting that higher MPV is also associated with increased arterial stiffness in females. This may be in contradiction with our previous work on sex specific determinants for MPV in males and females^17^, where we observed an association of MPV with CVRF in males, but not in females, where MPV was under strong influence of the female hormonal status. However, our previous study addressed the issue of MPV determinants in a wide age range including menstruating, premenopausal and postmenopausal women. Taking the increased incidence of cardiovascular events in females in the postmenopausal period into consideration, MPV might be determined by CVRFs in this group only, which could partly explain the positive association with arterial stiffness found in this study. Indeed, in menopausal women without hormone replacement therapy, diabetes, obesity and smoking were positively associated with MPV (see Supplemental Table 5).

The second important issue concerns the association of MPV-related genetic variants with arterial stiffness. Whereas the majority of studies focused on the association between arterial stiffness and SNPs related with the renin-angiotensin-aldosterone system^30^, the present studies investigated the association of SNPs related to MPV variability, as already described by GWAS and other works in the literature, with arterial stiffness. Four SNPs in males and one in females were related with AIx in a non-adjusted analysis. After adjustment for age, sex, CVRFs, comorbidities and medication in a multivariable logistic regression model, all observed associations were lost. This fact supports a potential causal relationship between MPV, arterial stiffness and the development of CVD, which needs to be further explored. Further analysis of these common genetic variants and total mortality revealed a strong relation of rs10076782 with increased mortality. Located on chromosome 5, rs10076782 is a genetic variant close to *Ring Finger Protein 145 gene (RNF145*), which is involved in megakaryopoiesis and platelet formation^20^. The precise link to arterial stiffness, however, remains still unclear.

Several limitations of this study need to be acknowledged. We did not apply correction for multiple testing for investigating the association of MPV-related genetic variants with arterial stiffness. Although this study was of explorative nature, it was, however, based on the hypothesis of a biological link between platelet activation and arterial stiffness. The hypothetical concept was confirmed by large multivariable models demonstrating a measurable clinical link in humans.

Life style changes in particular exercise and pharmacological interventions are two potential strategies suggested for decreasing arterial stiffness^31^. The association between MPV, as a marker of platelet reactivity, and arterial stiffness suggests a potential for antiplatelet agents in modulating arterial stiffness. Indeed, a recent study demonstrated that increased platelet reactivity even under clopidogrel medication is associated with impaired arterial stiffness in patients after percutaneous coronary intervention^32^. Furthermore, increased platelet reactivity was associated with adverse outcome in these patients. Therefore, platelets indeed represent an important player in the pathophysiology of increased arterial stiffness and therefore could act as potential therapeutic target.

In conclusion, with the present study we can demonstrate that MPV is closely and strongly associated with increased arterial stiffness in both males and females in the GHS population and we demonstrate for the first time potential joint genetic variants associated with both traits. Future investigations are necessary to fully understand the molecular processes how activated platelets interact with the vessel wall to cause increased vascular stiffness and what antiplatelet measures are necessary to improve arterial compliance.

## Additional Information

**How to cite this article**: Panova-Noeva, M. *et al*. Mean Platelet Volume and Arterial Stiffness – Clinical Relationship and Common Genetic Variability. *Sci. Rep.* **7**, 40229; doi: 10.1038/srep40229 (2017).

**Publisher's note:** Springer Nature remains neutral with regard to jurisdictional claims in published maps and institutional affiliations.

## Supplementary Material

Supplement Material

## Figures and Tables

**Table 1:** Sex-specific distribution of augmentation index, as marker of arterial stiffness according to quartiles of mean platelet volume (MPV).

		Total	Quartiles of Mean Platelet Volume				p for trend
			<25%	25–50%	50–75%	>75%	
Males	Age (y)	55.3 ± 11.1	54.0 ± 11.2	54.7 ± 11.1	55.7 ± 10.9	56.8 ± 11.0	<0.0001
	N	7571	2060	1833	1815	1863	—
	AIx (%)	11.38 ± 18.48	9.75 ± 17.90	10.34 ± 18.18	11.55 ± 18.54	14.05 ± 19.05	<0.0001
Females	Age (y)	54.7 ± 11.1	54.5 ± 10.9	54.8 ± 11.0	54.8 ± 11.2	54.9 ± 11.2	0.23
	N	7397	1829	1794	1925	1849	—
	AIx (%)	23.71 ± 20.59	22.40 ± 20.27	23.26 ± 20.40	24.08 ± 20.77	25.09 ± 20.83	<0.0001

N, number of individuals; AIx, augmentation index.

**Table 2:** Association of augmentation index and mean platelet volume (MPV) and/or platelet count – results from multivariable regression models.

Augmentation Index (%)	MPV (fL)				Platelet count (x10^9^/L)			
	Males		Females		Males		Females	
	β Estimate (95% CI)	P value	β Estimate (95% CI)	P value	β Estimate (95% CI)	P value	β Estimate (95% CI)	P value
**Model 1** Crude	1.79 (1.26; 2.32)	<0.0001	0.911 (0.311; 1.51)	0.0029	−0.011 (−0.019; −0.003)	0.0046	−0.009 (−0.017; −0.002)	0.015
**Model 2** Adjusted for age and cardiovascular risk factors	0.706 (0.250; 1.16)	0.0024	0.881 (0.328; 1.43)	0.0018	0.001 (−0.006; 0.007)	0.83	−0.004 (−0.012; 0.003)	0.22
**Model 3** Additionally adjusted for comorbidities	0.781 (0.317; 1.24)	0.00097	0.799 (0.238; 1.36)	0.0052	0.001 (−0.005; 0.008)	0.73	−0.004 (−0.011; 0.003)	0.27
**Model 4** Additionally adjusted for drug intake, fibrinogen, hematocrit	0.804 (0.335; 1.27)	0.00079	0.903 (0.337; 1.47)	0.0018	0.003 (−0.004; 0.009)	0.41	−0.001 (−0.009; 0.006)	0.72

Linear regression model with augmentation index in 5754 males and 5479 females as dependent variable. Results are beta estimates for change in augmentation index (%) with an increase in MPV of 1 fL (left) or increase in platelet count of 1 × 10^9^/l (right). Model 1: non-adjusted; Model 2: adjusted for age + cardiovascular risk factors (diabetes, obesity, hypertension, smoking, family history of myocardial infarction or stroke); Model 3: Model 2 + comorbidities (myocardial infarction, coronary artery disease, peripheral arterial disease, chronic heart failure, deep vein thrombosis, pulmonary embolism, stroke, atrial fibrillation, chronic kidney disease, chronic liver disease, chronic obstructive pulmonary disease, cancer, anemia, thyroidism, polyarthritis, autoimmune disease); Model 4: Model 3 + drug intake (antithrombotic, beta blockers, antilipemic, antihypertensive, angiotensin converting enzyme inhibitors, angiotensin receptor blockers, diuretics, calcium channel blockers) + fibrinogen + hematocrit.

**Table 3:** Association between MPV related genetic variants and arterial stiffness.

Males	Risk (Major) Allele	non-adjusted		adjusted for age and cardiovascular risk factors		additionally adjusted for age, cardiovascular risk factors, comorbidities and medication	
AIx [%]		β Estimate (95%CI)	p value	β Estimate (95%CI)	p value	β Estimate (95%CI)	p value
rs17397129	G (A)	2.12 (0.435; 3.81)	0.014	1.60 (0.114; 3.08)	0.035	1.22 (−0.299; 2.75)	0.12
rs7961894	T (C)	−2.19 (−4.01; −0.378)	0.018	−1.57 (−3.16; 0.028)	0.054	−1.52 (−3.15; 0.111)	0.068
rs10076782	A (G)	1.38 (0.116; 2.65)	0.032	0.625 (−0.488; 1.74)	0.27	0.607 (−0.529; 1.74)	0.29
rs7342306	A (G)	1.14 (0.019; 2.27)	0.046	0.842 (−0.144; 1.83)	0.094	0.836 (−0.170; 1.84)	0.10
**Females**							
rs11734132	G (C)	−1.90 (−3.74; −0.065)	0.042	−1.92 (−3.61; −0.217)	0.027	−1.68 (−3.41; 0.052)	0.057

Multivariable linear regression model for AIx (augmentation index) in males (n = 2033) and females (n = 1908). The beta estimates are the effect per one risk allele.

**Table 4:** Relation between MPV - related genetic variants and mortality.

SNPs	Alleles	HR (95% CI)	p-value
rs17397129	AG vs AA	0.73 (0.52; 1.01)	0.06
	GG vs AA	1.32 (0.54; 3.22)	0.53
rs7961894	TC vs CC	1.10 (0.80; 1.53)	0.53
	TT vs CC	1.36 (0.43; 4.27)	0.59
rs10076782	GA vs GG	1.37 (1.04; 1.81)	**0.024**
	AA vs GG	2.02 (1.35; 3.02)	**0.00061**
rs7342306	GA vs GG	1.02 (0.77; 1.35)	0.90
	AA vs GG	0.68 (0.45; 1.02)	0.065
rs11734132	GC vs CC	0.80 (0.59; 1.08)	0.15
	GG vs CC	1.37 (0.67; 2.78)	0.39

Cox-regression analysis for mortality (233 events) in 4175 individulals with adjustments for age and sex. The table is a summary of the cox-regression models tested for each genetic variant. P-values < 0.05 are printed in bold. HR, hazard ratio.
