# Supplementary material for: Mean Platelet Volume and Arterial Stiffness – Clinical Relationship and Common Genetic Variability
Source: Sci Rep. 2017 Jan 6;7:40229. doi: 10.1038/srep40229 (PMC5216402; doi:10.1038/srep40229)
Supplement: Supplement Material [file srep40229-s1.docx]

**Supplement material**

**Mean platelet volume and arterial stiffness –**

**Clinical relationship and common genetic variability**

Marina Panova-Noeva^1^, Natalie Arnold^2^, Iris Hermanns^1^, Jürgen H. Prochaska^1,2^, Andreas Schulz^3^, Henri M. Spronk^4^, Harald Binder^5^, Norbert Pfeiffer^6^, Manfred Beutel^7^, Stefan Blankenberg^8,9^ , Tanja Zeller^8,9^, Johannes Lotz^11^, Thomas Münzel^2,10^, Karl J. Lackner^10,11^, Hugo ten Cate^4^, Philipp S. Wild^1,3,10^

^1^ Center for Thrombosis and Hemostasis (CTH), University Medical Center of the Johannes Gutenberg-University Mainz, Germany

^2^ Center for Cardiology I, University Medical Center of the Johannes Gutenberg-University Mainz, Germany

^3^ Preventive Cardiology and Preventive Medicine, Center for Cardiology, University Medical Center of the Johannes Gutenberg-University Mainz, Germany

^4^ Laboratory for Clinical Thrombosis and Hemostasis, Department of Internal Medicine, Cardiovascular Research Institute Maastricht (CARIM), Maastricht University Medical Center, the Netherlands

^5^ Institute of Medical Biostatistics, Epidemiology and Informatics, University Medical Center of the Johannes Gutenberg-University Mainz, Germany

^6^ Department of Ophthalmology, University Medical Center of the Johannes Gutenberg-University Mainz, Germany

^7^ Department of Psychosomatic Medicine and Psychotherapy, University Medical Center of the Johannes Gutenberg-University Mainz, Germany

^8^ Department of General and Interventional Cardiology, University Heart Centre, Hamburg-Eppendorf, Germany

^9^ DZHK (German Center for Cardiovascular Research), Partner Site Hamburg/Kiel/Lübeck, Hamburg, Germany

^10^ DZHK (German Center for Cardiovascular Research), Partner Site RhineMain, Mainz, Germany

^11^ Institute for Clinical Chemistry and Laboratory Medicine, University Medical Center of the Johannes Gutenberg-University Mainz, Mainz, Germany

**Part A. Supplemental Methods**

**Definition of classical cardiovascular risk factors**

Obesity has been defined as body-mass index ≥ 30 kg/m². Individuals have been classified as smokers (daily smokers ≥1 cigarette/day and occasional smokers <1 cigarette/day) and non-smokers (former smokers and never smokers). Diabetes mellitus, dyslipidemia and hypertension have been defined in individuals with definite diagnose by a physician. In addition, diabetes mellitus has been defined if a blood glucose level of ≥126mg/dL in the baseline examination after an overnight fast of at least 8 hours or a blood glucose level of ≥200mg/dL in the baseline examination after a fasting period >5 hours. Dyslipidemia has been additionally defined if low-density lipoprotein/high-density lipoprotein ratio was >3.5 and/or triglycerides level ≥150mg/dl. Individuals taking antihypertensive drugs or having a mean systolic blood pressure of ≥140mmHg or a mean diastolic blood pressure of ≥90mmHg (in the 2nd and 3rd standardized measurement after 8 and 11 minutes of rest) have been also defined as present hypertension. Self-reported coronary artery disease (CAD), myocardial infarction (MI), heart failure (HF), stroke, deep vein thrombosis (DVT), pulmonary embolism (PE) and peripheral arterial disease (PAD) indicated personal history of cardiovascular disease. Positive family history was defined as at least one person with history of myocardial infarction and/or stroke as male first-degree relative until the age of 60 years or female first-degree relative until the age of 65 years.

**Categorization of medication**

Medications taken by study participants were registered on site at the GHS study center by scanning bar codes from the drug packages. The medication was classified according to the Anatomical Therapeutic Chemical (ATC) classification system. The following medication groups were selected for analysis: antithrombotic agents (B01A), aspirin (B01AC56), antilipemic drugs (C10A), antihypertensive drugs (C02), angiotensin converting enzyme (ACE) inhibitor (C09), diuretics (C03), angiotensin receptor blocker (ARBs) (C09C), calcium channel blocker (C08) and B-blocker (C07). For the use of oral contraceptives and/or hormone replacement therapy self-reported information was used.

**Part B. Supplemental Tables**

**Table 1. SNPs associated with MPV evaluated in the present analysis on the GHS population sample**

| **SNP in GWAS** | **Chromosome:Position** | **Gene** | **First reference publication** | **Tag SNP in Affym. 6.0 with r^2^ >0.8** | **r^2^  between lead SNP and tag SNP** | **Distance** |
| --- | --- | --- | --- | --- | --- | --- |
| rs12485738 | 3:56831748 | *ARHGEF3* | Meisinger C. et al. Am J Hum Genet. 2009 | na | na | na |
| rs7961894 | 12:121927677 | *WDR66* | Meisinger C. et al. Am J Hum Genet. 2009 | na | na | na |
| rs2138852 | 17:29376331 | *TAOK1* | Meisinger C. et al. Am J Hum Genet. 2009 | na | na | na |
| rs342293 | 7:106731773 | *FLJ36031; PIC3CG* | Soranzo N et al. Blood 2009 | na | na | na |
| rs10506328 | 12:54293448 | *NFE2; COPZ1* | Soranzo N et al. Blood 2009 | na | na | na |
| rs1668873 | 1:205266862 | *TMCC2* | Soranzo N et al. Nat Genet 2009 | rs1172111 | 1.0 | 3010 |
| rs11071720 | 15:63049797 | *TPM1* | Soranzo N et al. Nat Genet 2009 | rs4774471 | 0.824 | 9351 |
| rs6136489 | 20:1943088 | *SIPRA* | Soranzo N et al. Nat Genet 2009 | rs4814776 | 1.0 | 2211 |
| rs6110278 | 20:14407569 | *MACROD2* | Soranzo N et al. Nat Genet 2009 | rs6135137 | 1.0 | 15903 |
| rs17396340 | 1:10226118 | *KIF1B; MIR1273D* | Gieger C. et al. Nature 2011 | rs17397129 | 1.0 | 59286 |
| rs1172130 | 1:205275825 | *TMCC2* | Gieger C. et al. Nature 2011 | rs4951184 | 0.932 | 4294 |
| rs649729 | 2:31241519 | *EHD3* | Gieger C. et al. Nature 2011 | na | na | na |
| rs4305276 | 2:240555596 | *ANKMY1* | Gieger C. et al. Nature 2011 | rs4676428 | 0.964 | 2758 |
| rs10512627 | 3:124621375 | *KALRN* | Gieger C. et al. Nature 2011 | rs10512628 | 0.967 | 1584 |
| rs11734132 | 4:6889792 | *KIAA0232* | Gieger C. et al. Nature 2011 | na | na | na |
| rs2227831 | 5:76727669 | *F2R* | Gieger C. et al. Nature 2011 | rs17568628 | 1.0 | 23445 |
| rs4521516 | 5:88804134 | *MEF2C* | Gieger C. et al. Nature 2011 | na | na | na |
| rs10076782 | 5:159177955 | *RNF145* | Gieger C. et al. Nature 2011 | na | na | na |
| rs10813766 | 9:331490 | *DOCK8* | Gieger C. et al. Nature 2011 | rs2296828 | 1.0 | 3484 |
| rs17655730 | 11:270715 | *PSMD13; NLRP6* | Gieger C. et al. Nature 2011 | na | na | na |
| rs1558324 | 12:6180053 | *CD9; VWF* | Gieger C. et al. Nature 2011 | rs7342306 | 1.0 | 1874 |
| rs10876550 | 12:54318524 | *COPZ1, NFE2, CBX5* | Gieger C. et al. Nature 2011 | na | na | na |
| rs2950390 | 12:56661507 | *PTGES3; BAZ2A* | Gieger C. et al. Nature 2011 | na | na | na |
| rs7317038 | 13:113358583 | *GRTP1; GRTP1-AS1* | Gieger C. et al. Nature 2011 | na | na | na |
| rs944002 | 14:103106478 | *C14orf73* | Gieger C. et al. Nature 2011 | rs2297067 | 1.0 | 6030 |
| rs3000073 | 14:105263455 | *BRF1* | Gieger C. et al. Nature 2011 | rs2816608 | 1.0 | 11385 |
| rs8076739 | 17:29387569 | *TAOK1* | Gieger C. et al. Nature 2011 | rs2138852 | 1.0 | 11238 |
| rs16971217 | 17:35617036 | *SNORD7‐ AP2B1* | Gieger C. et al. Nature 2011 | rs10512472 | 1.0 | 59251 |
| rs12969657 | 18:69869260 | *CD226* | Gieger C. et al. Nature 2011 | na | na | na |
| rs13042885 | 20:1944061 | *SIPRA* | Gieger C. et al. Nature 2011 | na | na | na |
| rs4812048 | 20:59012716 | *CTSZ; TUBB1* | Gieger C. et al. Nature 2011 | na | na | na |
| rs2180748 | 1:171977878 | *DNM3* | Shameer K. et al. Hum Genet 2014 | na | na | na |
| rs342240 | 7:106696804 | *FLJ36031-PIK3CG* | Shameer K. et al. Hum Genet. 2014 | rs342251 | 1.0 | 7559 |
| rs4379723 | 10:63203689 | *JMJD1C* | Shameer K. et al. Hum Genet. 2014 | rs12355784 | 1.0 | 158116 |
| rs7075195 | 10:63290899 | *JMJD1C; NRBF2; REEP3* | Shameer K. et al. Hum Genet. 2014 | rs10761741 | 0.902 | 15527 |
| rs9900280 | 17:29442580 | *TAOK1* | Shameer K. et al. Hum Genet. 2014 | rs8081267 | 1.0 | 5688 |
| rs6687605 | 1:25563141 | *LDLRAP1* | Erdmann J. et al. Am J Hum Genet. 2016 | na | na | na |
| rs56043070 | 1:247556467 | *GCSAML* | Erdmann J. et al. Am J Hum Genet. 2016 | na | na | na |
| rs1339847 | 1:247875992 | *TRIM58* | Erdmann J. et al. Am J Hum Genet. 2016 | na | na | na |
| rs34950321 | 5:76668682 | *IQGAP2* | Erdmann J. et al. Am J Hum Genet. 2016 | na | na | na |
| rs34592828 | 5:76701084 | *IQGAP2* | Erdmann J. et al. Am J Hum Genet. 2016 | na | na | na |
| rs1012899 | 6:25604863 | *LRRC16A* | Erdmann J. et al. Am J Hum Genet. 2016 | na | na | na |
| rs664370 | 6:36426039 | *PXT1* | Erdmann J. et al. Am J Hum Genet. 2016 | na | na | na |
| rs2343596 | 8:105580979 | *ZFPM2* | Erdmann J. et al. Am J Hum Genet. 2016 | na | na | na |
| rs55895668 | 8:143926863 | *PLEC* | Erdmann J. et al. Am J Hum Genet. 2016 | na | na | na |
| rs4909945 | 11:10652192 | *MRVI1* | Erdmann J. et al. Am J Hum Genet. 2016 | na | na | na |
| rs11125 | 14:55145121 | *LGALS3* | Erdmann J. et al. Am J Hum Genet. 2016 | na | na | na |
| rs2010875 | 15:64865283 | *PLEKHO2* | Erdmann J. et al. Am J Hum Genet. 2016 | na | na | na |
| rs35385129 | 19:44658921 | *PVR* | Erdmann J. et al. Am J Hum Genet. 2016 | na | na | na |
| rs2243603 | 20:1566265 | *SIRPB1* | Erdmann J. et al. Am J Hum Genet. 2016 | na | na | na |
| rs1018448 | 22:42810944 | *ARFGAP3* | Erdmann J. et al. Am J Hum Genet. 2016 | na | na | na |

na: not applicable

**Table 2. Sex-specific associations of MPV-related genetic variants and augmentation index**

**2A.**

| **Males** | **non-adjusted** | | **adjusted*** | | **adjusted**** | |
| --- | --- | --- | --- | --- | --- | --- |
| **AIx (%)** | **Beta (95% CI)** | **p-value** | **Beta (95% CI)** | **p-value** | **Beta (95% CI)** | **p-value** |
| rs342293 | -0.126 (-1.26; 1.01) | 0.83 | -0.34 (-1.33; 0.65) | 0.50 | -0.146 (-1.16; 0.87) | 0.78 |
| rs10506328 | -0.960 (-2.12; 0.201) | 0.11 | -0.78 (-1.80; 0.238) | 0.13 | -0.801 (-1.84; 0.239) | 0.13 |
| rs7961894 | -2.19 (-4.01; -0.378) | **0.018** | -1.57 (-3.16; 0.028) | 0.054 | -1.52 (-3.15; 0.111) | 0.068 |
| rs12485738 | -0.589 (-1.77; 0.588) | 0.33 | -0.616 (-1.65; 0.418) | 0.24 | -0.621 (-1.68; 0.434) | 0.25 |
| rs2138852 | -0.133 (-1.25; 0.983) | 0.82 | 0.363 (-0.617; 1.34) | 0.47 | 0.307 (-0.697; 1.31) | 0.55 |
| rs2180748 | 0.235 (-0.924; 1.39) | 0.69 | 0.067 (-0.953; 1.09) | 0.90 | 0.272 (-0.77; 1.31) | 0.61 |
| rs10076782 | 1.38 (0.116; 2.65) | **0.032** | 0.625 (-0.488; 1.74) | 0.27 | 0.607 (-0.529; 1.74) | 0.29 |
| rs10876550 | -1.05 (-2.20; 0.0947) | 0.072 | -1.03 (-2.04; -0.022) | **0.045** | -1.16 (-2.19; -0.131) | **0.027** |
| rs11734132 | 0.174 (-1.50; 1.85) | 0.84 | -0.089 (-1.56; 1.38) | 0.90 | -0.529 (-2.03; 0.975) | 0.49 |
| rs12969657 | 0.234 (-0.930; 1.40) | 0.69 | 0.262 (-0.761; 1.28) | 0.62 | 0.193 (-0.856; 1.24) | 0.72 |
| rs13042885 | 0.610 (-0.651; 1.87) | 0.34 | 0.351 (-0.757; 1.46) | 0.53 | 0.381 (-0.751; 1.51) | 0.51 |
| rs17655730 | -0.889 (-2.25; 0.475) | 0.20 | -0.635 (-1.83; 0.562) | 0.30 | -0.377 (-1.60; 0.85) | 0.55 |
| rs2950390 | -0.660 (-1.86; 0.539) | 0.28 | -0.359 (-1.41; 0.696) | 0.50 | -0.324 (-1.41; 0.758) | 0.56 |
| rs4521516 | 0.802 (-0.967; 2.57) | 0.37 | -0.194 (-1.75; 1.36) | 0.81 | -0.354 (-1.95; 1.24) | 0.66 |
| rs4812048 | 0.743 (-0.816; 2.30) | 0.35 | 0.929 (-0.44; 2.30) | 0.18 | 0.794 (-0.60; 2.19) | 0.26 |
| rs649729 | -1.15 (-2.52; 0.228) | 0.10 | -0.744 (-1.95; 0.465) | 0.23 | -0.846 (-2.09; 0.394) | 0.18 |
| rs7317038 | 0.0370 (-1.18; 1.25) | 0.95 | 0.113 (-0.955; 1.18) | 0.84 | 0.136 (-0.963; 1.24) | 0.81 |
| rs342251 | -0.0639 (-1.20; 1.08) | 0.91 | -0.327 (-1.33; 0.675) | 0.52 | -0.158 (-1.18; 0.865) | 0.76 |
| rs12355784 | -0.371 (-1.50; 0.756) | 0.52 | -0.833 (-1.82; 0.158) | 0.099 | -0.87 (-1.88; 0.141) | 0.092 |
| rs8081267 | -0.0622 (-1.18; 1.06) | 0.91 | 0.431 (-0.555; 1.42) | 0.39 | 0.386 (-0.624; 1.40) | 0.45 |
| rs10761741 | -0.374 (-1.51; 0.764) | 0.52 | -0.76 (-1.76; 0.241) | 0.14 | -0.817 (-1.84; 0.204) | 0.12 |
| rs6070696 | 0.693 (-0.849; 2.23) | 0.38 | 0.888 (-0.464; 2.24) | 0.20 | 0.725 (-0.65; 2.10) | 0.30 |
| rs4951184 | -1.04 (-2.21; 0.127) | 0.080 | -0.77 (-1.80; 0.259) | 0.14 | -0.858 (-1.91; 0.189) | 0.11 |
| rs7342306 | 1.14 (0.0192; 2.27) | **0.046** | 0.842 (-0.144; 1.83) | 0.094 | 0.836 (-0.17; 1.84) | 0.10 |
| rs17568628 | -0.519 (-3.63; 2.59) | 0.74 | -0.912 (-3.64; 1.82) | 0.51 | -1.04 (-3.87; 1.80) | 0.47 |
| rs2296828 | -0.320 (-1.55; 0.913) | 0.61 | -0.208 (-1.29; 0.876) | 0.71 | -0.389 (-1.49; 0.713) | 0.49 |
| rs10512472 | 0.173 (-1.33; 1.68) | 0.82 | 0.015 (-1.31; 1.34) | 0.98 | 0.0926 (-1.25; 1.44) | 0.89 |
| rs4676428 | 0.203 (-1.04; 1.44) | 0.75 | 0.527 (-0.565; 1.62) | 0.34 | 0.617 (-0.494; 1.73) | 0.28 |
| rs2816608 | -0.214 (-1.48; 1.05) | 0.74 | 0.398 (-0.712; 1.51) | 0.48 | 0.509 (-0.62; 1.64) | 0.38 |
| rs2297067 | -0.963 (-2.29; 0.361) | 0.15 | -0.241 (-1.41; 0.923) | 0.68 | -0.476 (-1.67; 0.715) | 0.43 |
| rs10512628 | -0.106 (-1.23; 1.02) | 0.85 | -0.451 (-1.43; 0.532) | 0.37 | -0.597 (-1.60; 0.409) | 0.25 |
| rs17397129 | 2.12 (0.435; 3.81) | **0.014** | 1.60 (0.114; 3.08) | **0.035** | 1.22 (-0.299; 2.75) | 0.12 |
| rs1172111 | -1.03 (-2.20; 0.145) | 0.086 | -0.755 (-1.78; 0.274) | 0.15 | -0.839 (-1.89; 0.209) | 0.12 |
| rs4814776 | 0.578 (-0.610; 1.77) | 0.34 | 0.486 (-0.557; 1.53) | 0.36 | 0.403 (-0.66; 1.47) | 0.46 |
| rs4774471 | 0.358 (-0.862; 1.58) | 0.57 | 0.131 (-0.94; 1.20) | 0.81 | 0.249 (-0.846; 1.34) | 0.66 |
| rs6135137 | 1.05 (-0.214; 2.32) | 0.10 | 0.527 (-0.587; 1.64) | 0.35 | 0.468 (-0.669; 1.60) | 0.42 |
| rs6687605 | -0.114 (-1.29; 1.06) | 0.85 | -0.057(-1.09; 0.973) | 0.91 | -0.252 (-1.31; 0.804) | 0.64 |
| rs56043070 | -2.58 (-5.59; 0.43) | 0.093 | -0.921 (-3.57; 1.73) | 0.50 | -0.741 (-3.45; 1.96) | 0.59 |
| rs1339847 | -0.878 (-2.95; 1.20) | 0.41 | -0.544 (-2.37; 1.28) | 0.56 | -0.70 (-2.55; 1.15) | 0.46 |
| rs34950321 | 2.10 (-2.42; 6.63) | 0.36 | 1.91 (-2.06; 5.87) | 0.35 | 2.29 (-1.74; 6.33) | 0.27 |
| rs34592828 | -0.193 (-3.29; 2.90) | 0.90 | -0.146 (-2.86; 2.57) | 0.92 | -0.197 (-3.01; 2.61) | 0.89 |
| rs1012899 | 0.72 (-0.626; 2.07) | 0.29 | 0.598 (-0.584; 1.78) | 0.32 | 0.42 (-0.785; 1.63) | 0.49 |
| rs664370 | 0.252 (-0.976; 1.48) | 0.69 | 0.118 (-0.96; 1.20) | 0.83 | 0.479 (-0.624; 1.58) | 0.39 |
| rs2343596 | -0.49 (-1.80; 0.824) | 0.46 | 0.065 (-1.09; 1.22) | 0.91 | 0.104 (-1.08; 1.29) | 0.86 |
| rs55895668 | -0.55 (-1.77; 0.66) | 0.37 | -0.139 (-1.20; 0.927) | 0.80 | -0.208 (-1.29; 0.878) | 0.71 |
| rs4909945 | 0.979 (-0.19; 2.15) | 0.10 | 0.439 (-0.594; 1.47) | 0.41 | 0.272 (-0.78; 1.32) | 0.61 |
| rs11125 | -1.52 (-3.58; 0.54) | 0.15 | -1.03 (-2.85; 0.777) | 0.26 | -1.26 (-3.12; 0.595) | 0.18 |
| rs2010875 | 0.258 (-1.35; 1.86) | 0.75 | 0.64 (-0.77; 2.05) | 0.37 | 0.597 (-0.843; 2.04) | 0.42 |
| rs35385129 | 0.39 (-1.37; 2.15) | 0.66 | 0.58 (-0.963; 2.12) | 0.46 | 0.512 (-1.06; 2.09) | 0.52 |
| rs2243603 | 0.76 (-0.61; 2.14) | 0.28 | 0.107 (-1.10; 1.32) | 0.86 | 0.0943 (-1.15; 1.34) | 0.88 |
| rs1018448 | 0.72 (-0.393; 1.83) | 0.20 | 0.328 (-0.65; 1.31) | 0.51 | 0.288 (-0.709; 1.28) | 0.57 |

2B.

| **Females** | **non-adjusted** | | **adjusted*** | | **adjusted**** | |
| --- | --- | --- | --- | --- | --- | --- |
| **AIx (%)** | **Beta (95% CI)** | **p-value** | **Beta (95% CI)** | **p-value** | **Beta (95% CI)** | **p-value** |
| rs342293 | -0.407 (-1.67; 0.857) | 0.53 | -0.368 (-1.54; 0.801) | 0.54 | -0.356 (-1.55; 0.839) | 0.56 |
| rs10506328 | -0.495 (-1.78; 0.793) | 0.45 | -0.502 (-1.69; 0.687) | 0.41 | -0.641 (-1.85; 0.573) | 0.30 |
| rs7961894 | -0.823 (-2.86; 1.21) | 0.43 | -0.148 (-2.03; 1.73) | 0.88 | 0.0409 (-1.86; 1.95) | 0.97 |
| rs12485738 | 0.830 (-0.48; 2.14) | 0.21 | 0.947 (-0.263; 2.16) | 0.12 | 1.05 (-0.179; 2.27) | 0.094 |
| rs2138852 | 1.16 (-0.091; 2.40) | 0.069 | 1.02 (-0.131; 2.17) | 0.082 | 1.15 (-0.0215; 2.32) | 0.054 |
| rs2180748 | 0.782 (-0.498; 2.06) | 0.23 | 0.951 (-0.23; 2.13) | 0.11 | 0.984 (-0.211; 2.18) | 0.11 |
| rs10076782 | -0.0083 (-1.41; 1.39) | 0.99 | -0.131 (-1.42; 1.16) | 0.84 | -0.084 (-1.40; 1.23) | 0.90 |
| rs10876550 | -0.598 (-1.85; 0.657) | 0.35 | -0.526 (-1.69; 0.635) | 0.37 | -0.597 (-1.78; 0.587) | 0.32 |
| rs11734132 | -1.90 (-3.74; -0.065) | **0.042** | -1.92 (-3.61; -0.217) | **0.027** | -1.68 (-3.41; 0.0515) | 0.057 |
| rs12969657 | -0.193 (-1.47; 1.09) | 0.77 | -0.0534 (-1.24; 1.13) | 0.93 | -0.119 (-1.33; 1.09) | 0.85 |
| rs13042885 | -0.923 (-2.35; 0.509) | 0.21 | -0.751 (-2.07; 0.572) | 0.27 | -0.658 (-2.00; 0.688) | 0.34 |
| rs17655730 | 0.106 (-1.41; 1.63) | 0.89 | 0.659 (-0.745; 2.06) | 0.36 | 0.746 (-0.694; 2.19) | 0.31 |
| rs2950390 | 0.346 (-0.979; 1.67) | 0.61 | 0.181 (-1.04; 1.40) | 0.77 | 0.291 (-0.956; 1.54) | 0.65 |
| rs4521516 | 0.281 (-1.63; 2.19) | 0.77 | 0.179 (-1.59; 1.94) | 0.84 | 0.0321 (-1.77; 1.83) | 0.97 |
| rs4812048 | -1.02 (-2.73; 0.70) | 0.25 | -0.366 (-1.95; 1.22) | 0.65 | -0.492 (-2.10; 1.12) | 0.55 |
| rs649729 | -0.898 (-2.45; 0.658) | 0.26 | -0.873 (-2.31; 0.565) | 0.23 | -1.05 (-2.51; 0.399) | 0.16 |
| rs7317038 | 1.01 (-0.41; 2.42) | 0.16 | 0.91 (-0.397; 2.22) | 0.17 | 0.782 (-0.556; 2.12) | 0.25 |
| rs342251 | -0.407 (-1.68; 0.87) | 0.53 | -0.379 (-1.56; 0.801) | 0.53 | -0.346 (-1.55; 0.858) | 0.57 |
| rs12355784 | 0.447 (-0.82; 1.71) | 0.49 | -0.185 (-1.36; 0.989) | 0.76 | -0.0301 (-1.22; 1.16) | 0.96 |
| rs8081267 | 1.16 (-0.0986; 2.41) | 0.071 | 1.00 (-0.16; 2.16) | 0.091 | 1.12 (-0.0567; 2.30) | 0.062 |
| rs10761741 | 0.346 (-0.932; 1.62) | 0.60 | -0.344 (-1.53; 0.84) | 0.57 | -0.236 (-1.44; 0.966) | 0.70 |
| rs6070696 | -0.917 (-2.62; 0.783) | 0.29 | -0.222 (-1.79; 1.35) | 0.78 | -0.411 (-2.00; 1.18) | 0.61 |
| rs4951184 | -0.104 (-1.38; 1.17) | 0.87 | -0.256 (-1.43; 0.918) | 0.67 | -0.206 (-1.40; 0.986) | 0.73 |
| rs7342306 | 0.550 (-0.752; 1.85) | 0.41 | 0.695 (-0.508; 1.90) | 0.26 | 0.862 (-0.361; 2.08) | 0.17 |
| rs17568628 | 0.124 (-3.49; 3.74) | 0.95 | -0.346 (-3.69; 2.99) | 0.84 | -0.678 (-4.08; 2.73) | 0.70 |
| rs2296828 | 0.542 (-0.828; 1.91) | 0.44 | 0.870 (-0.395; 2.14) | 0.18 | 0.619 (-0.673; 1.91) | 0.35 |
| rs10512472 | 1.36 (-0.282; 3.00) | 0.10 | 1.17 (-0.339; 2.69) | 0.13 | 0.992 (-0.544; 2.53) | 0.21 |
| rs4676428 | -0.132 (-1.48; 1.22) | 0.85 | -0.234 (-1.48; 1.01) | 0.71 | -0.294 (-1.56; 0.972) | 0.65 |
| rs2816608 | 0.572 (-0.831; 1.97) | 0.42 | 0.251 (-1.04; 1.55) | 0.70 | 0.0812 (-1.24; 1.40) | 0.90 |
| rs2297067 | -0.597 (-2.14; 0.945) | 0.45 | -0.522 (-1.95; 0.903) | 0.47 | -0.652 (-2.10; 0.795) | 0.38 |
| rs10512628 | 0.022 (-1.25; 1.30) | 0.97 | 0.215 (-0.963; 1.39) | 0.72 | -0.056 (-1.26; 1.15) | 0.93 |
| rs17397129 | 0.478 (-1.40; 2.36) | 0.62 | 0.17 (-1.57; 1.91) | 0.85 | 0.143 (-1.62; 1.91) | 0.87 |
| rs1172111 | -0.131 (-1.40; 1.14) | 0.84 | -0.289 (-1.46; 0.884) | 0.63 | -0.242 (-1.43; 0.95) | 0.69 |
| rs4814776 | -0.429 (-1.78; 0.917) | 0.53 | -0.333 (-1.58; 0.911) | 0.60 | -0.258 (-1.52; 1.01) | 0.69 |
| rs4774471 | 0.063 (-1.27; 1.40) | 0.93 | 0.674 (-0.56; 1.91) | 0.28 | 0.66 (-0.591; 1.91) | 0.30 |
| rs6135137 | -0.847 (-2.23; 0.534) | 0.23 | -0.354 (-1.63; 0.922) | 0.59 | -0.394 (-1.69; 0.901) | 0.55 |
| rs6687605 | 0.488 (-0.854; 1.83) | 0.48 | 0.112 (-1.13; 1.35) | 0.86 | -0.105 (-1.36; 1.15) | 0.87 |
| rs56043070 | -2.00 (-5.50; 1.49) | 0.26 | -1.02 (-4.25; 2.21) | 0.54 | -1.12 (-4.37; 2.14) | 0.50 |
| rs1339847 | -1.07 (-3.36; 1.21) | 0.36 | -0.643 (-2.75; 1.47) | 0.55 | -0.909 (-3.05; 1.23) | 0.40 |
| rs34950321 | 0.761 (-4.16; 5.68) | 0.76 | -0.0332 (-4.58; 4.51) | 0.99 | 0.0486 (-4.57; 4.66) | 0.98 |
| rs34592828 | 0.023 (-3.56; 3.60) | 0.99 | -0.141 (-3.45; 3.17) | 0.93 | -0.426 (-3.78; 2.93) | 0.80 |
| rs1012899 | -0.319 (-1.84; 1.20) | 0.68 | 0.0029 (-1.40; 1.40) | 1.00 | 0.00617 (-1.42; 1.44) | 0.99 |
| rs664370 | 0.413 (-0.927; 1.75) | 0.55 | 0.450 (-0.787; 1.69) | 0.48 | 0.270 (-0.995; 1.53) | 0.68 |
| rs2343596 | 0.330 (-1.10; 1.77) | 0.65 | 0.381 (-0.944; 1.71) | 0.57 | 0.305 (-1.04; 1.65) | 0.66 |
| rs55895668 | 0.332 (-1.04; 1.70) | 0.63 | 0.429 (-0.832; 1.69) | 0.50 | 0.297 (-0.98; 1.58) | 0.65 |
| rs4909945 | -0.014 (-1.37; 1.34) | 0.98 | 0.253 (-0.996; 1.50) | 0.69 | 0.621 (-0.65; 1.89) | 0.34 |
| rs11125 | 2.12 (-0.147; 4.39) | 0.067 | 1.49 (-0.607; 3.59) | 0.16 | 1.32 (-0.804; 3.45) | 0.22 |
| rs2010875 | -0.791 (-2.53; 0.94) | 0.37 | -0.649 (-2.25; 0.957) | 0.43 | -0.883 (-2.52; 0.749) | 0.29 |
| rs35385129 | -0.765 (-2.70; 1.17) | 0.44 | -0.989 (-2.78; 0.804) | 0.28 | -1.24 (-3.08; 0.587) | 0.18 |
| rs2243603 | -0.417 (-1.99; 1.15) | 0.60 | -0.44 (-1.89; 1.01) | 0.55 | -0.635 (-2.11; 0.842) | 0.40 |
| rs1018448 | -1.09 (-2.34; 0.168) | 0.090 | -0.612 (-1.77; 0.549) | 0.30 | -0.511 ( -1.70; 0.675) | 0.40 |

Multivariable linear regression model for augmentation index in males (2A, n = 2033) and females (2B, n = 1908).*adjusted for age and cardiovascular risk factors; **adjusted for age, cardiovascular risk factors, comorbidities and medication. P-Values <0.05 are printed in bold.

**Table 3. Relation between MPV, AIx and mortality**

|  | **HR (95% CI)** | **p-value** |
| --- | --- | --- |
| Sex | 0.42 (0.32; 0.56) | **< 0.0001** |
| Age (5 years) | 1.62 (1.52; 1.73) | **< 0.0001** |
| MPV (per SD) | 1.14 (1.03; 1.25) | **0.0082** |
| AIx (per SD) | 1.25 (1.11; 1.39) | **0.00016** |
| HR (bpm) | 1.03 (1.02; 1.04) | **< 0.0001** |
| Height (cm) | 0.99 (0.98; 1.01) | 0.20 |
| AI*MPV | 0.94 (0.86; 1.04) | 0.22 |

Cox regression analysis for mortality (428 events) in 11,185 individuals with adjustment for all variables in the table. All continuous variables were centered to the respective means. P-values <0.05 are printed in bold. HR, hazard ratio; MPV, mean platelet volume; AIx, augmentation index; HR, heart rate; bpm, beats per minute; SD, Standard Deviation.

**Table 4. Determinants of MPV in postmenopausal women**

| **MPV (fL)** | **Beta (L 95% CI; U 95% CI)** | **p-value** |
| --- | --- | --- |
| **Age (years)** | 0.00167 (-0.00184; 0.00517) | 0.35 |
| **Hypertension** | 0.0329 (-0.0272; 0.0931) | 0.28 |
| **Diabetes** | 0.193 (0.0876; 0.299) | **0.00034** |
| **Dyslipidemia** | -0.0528 (-0.115; 0.00894) | 0.094 |
| **Obesity** | 0.0690 (0.00604; 0.132) | **0.032** |
| **Smoking** | 0.134 (0.0577; 0.210) | **0.00057** |
| **FH of MI/Stroke** | -0.0799 (-0.143; -0.0168) | **0.013** |

Multivariable linear regression analysis including 4187 postmenopausal women without hormone replacement therapy. R^2^=0.0082; MPV, mean platelet volume; FH of MI/Stroke, Family history of myocardial infarction or stroke.
